# Effects of intracranial pressure monitoring in pediatric severe traumatic brain injury: a meta-analysis of cohort studies

**DOI:** 10.3389/fneur.2025.1557820

**Published:** 2025-03-17

**Authors:** Shan Xue, Zhe Zhang, Yan Liu

**Affiliations:** ^1^Department of Critical Care Medicine, West China Hospital, Sichuan University, Chengdu, China; ^2^West China School of Nursing, Sichuan University, Chengdu, China; ^3^Department of Neurosurgery, West China Hospital, Sichuan University, Chengdu, China

**Keywords:** intracranial pressure monitoring, severe traumatic brain injury, pediatric, mortality, complications, meta-analysis

## Abstract

**Introduction:**

As Severe traumatic brain injury (TBI) is a major cause of pediatric morbidity and mortality. The clinical benefits of intracranial pressure (ICP) monitoring in pediatric TBI remain debated. This meta-analysis aims to assess the impact of ICP monitoring on outcomes in children with severe TBI.

**Methods:**

Following PRISMA guidelines, a comprehensive search was conducted in PubMed, EMBASE, Cochrane Library, and Web of Science. Studies comparing pediatric severe TBI patients with and without ICP monitoring were included. Primary outcomes included in-hospital mortality and complications, while secondary outcomes included craniotomy/craniectomy rate, length of hospital stay and ICU stay, mechanical ventilation duration, and medical costs. Quality assessment was performed using the Methodological Index for Non-Randomized Studies (MINORS) for cohort studies. The weighted mean difference (WMD) for continuous variables and odds ratio (OR) for dichotomous variables were calculated, along with 95% confidence intervals (CIs). Meta-analysis was performed using RevMan 5.4.1 software.

**Results:**

Eight studies (12,987 patients) were included. ICP monitoring showed no significant impact on overall in-hospital mortality (OR, 1.14; *p* = 0.65), though propensity score matching (PSM) studies indicated a lower mortality rate with ICP monitoring (OR, 0.62; *p* = 0.005). However, ICP monitoring was associated with higher risks of infection-related (OR, 7.21; *p* < 0.001) and respiratory complications (OR, 5.79; *p* < 0.001), thromboembolic events (OR, 5.37; *p* < 0.001), increased craniotomy/craniectomy rates (OR, 2.34; *P* = 0.01), longer hospital (OR, 12.00; *p* < 0.001) and ICU stays (OR, 7.82; *p* < 0.001), extended mechanical ventilation durations (OR, 5.82; *p* < 0.001), and higher medical costs (WMD, 10.49; *p* = 0.006).

**Conclusion:**

This meta-analysis found no overall reduction in in-hospital mortality with ICP monitoring in pediatric severe TBI, potentially due to baseline severity imbalances in retrospective studies. However, PSM studies suggest a mortality benefit, indicating that ICP monitoring may be effective when confounding is minimized. Increased complication risks, longer hospital/ICU stays, prolonged ventilation, and higher costs were associated with monitoring, though these may reflect injury severity rather than monitoring itself. Given the limitations of this study, these findings should be interpreted cautiously.

## 1 Introduction

Severe traumatic brain injury (TBI) is a major cause of morbidity and mortality among children globally, with an estimated incidence ranging from 47 to 280 cases per 100,000 children (1, 2). While high-income countries report higher annual TBI incidence rates, the overall burden is disproportionately greater in low-income countries (3). Elevated intracranial pressure (ICP), a major complication of severe TBI, occurs due to changes in intracranial volume from the initial injury and secondary factors that increase cerebral blood volume, disrupting brain perfusion and raising the risk of herniation (4, 5).

Elevated ICP in children with severe TBI is strongly associated with poor outcomes (6–10). Even slight increases in ICP can reduce cerebral perfusion pressure and blood flow, leading to hypoxia and ischemia (8). The management of elevated ICP is widely regarded as a cornerstone of modern treatment for pediatric severe TBI (11). ICP monitoring plays a vital role in this process, providing real-time data on intracranial dynamics to guide therapeutic interventions. Guidelines recommend ICP monitoring for children with severe TBI, defined by a Glasgow Coma Scale (GCS) score of 8 or less, especially when imaging studies reveal abnormalities (7, 11, 12). Common monitoring methods include intraventricular catheters and parenchymal monitors, each suited to specific clinical scenarios (8).

The evidence for ICP monitoring in pediatric severe TBI remains highly controversial, with limited level I or II evidence available (8). Some studies suggest that ICP monitoring may reduce mortality and improve functional outcomes (13–15), while others report no significant benefit or even potential harm (16–20). To date, only one randomized controlled trial (RCT) has specifically addressed ICP monitoring in severe TBI, finding no outcome differences between monitored and empiric treatment groups (21). However, this study included only patients aged 12 years and older. Additional challenges include the invasiveness of the procedure, risk of complications, increased resource utilization, and variability in ICP management strategies across institutions, all of which contribute to the ongoing debate (8). The lack of a systematic review specifically addressing ICP monitoring in pediatric severe TBI underscores the need for a comprehensive meta-analysis to clarify its role in this population.

This meta-analysis aims to evaluate the effects of ICP monitoring in children with severe TBI. We hypothesize that ICP monitoring may provide benefits in this population, potentially improving survival rates and guiding more effective management strategies.

## 2 Methods

### 2.1 Literature search

This meta-analysis was conducted following the Preferred Reporting Items for Systematic Reviews and Meta-Analyses (PRISMA) guidelines (22). A comprehensive search of PubMed, EMBASE, the Cochrane Library, and Web of Science databases was performed independently by two reviewers, encompassing all records up to December 15, 2024. The primary search strategy included the following terms: (“Intracranial Pressure Monitoring” OR “ICP monitoring” OR “ICP measurement”) AND (“traumatic brain injury” OR “TBI” OR “severe head injury” OR “brain injury”) AND (“children” OR “pediatric” OR “child”) AND (“outcomes” OR “mortality” OR “complications” OR “neurological outcomes” OR “functional recovery” OR “prognosis”). The search syntax was tailored to meet the specific requirements of each database. Any discrepancies that arose during the search were addressed through discussions with a third reviewer.

### 2.2 Selection criteria

Inclusion criteria: (1) studies focusing on pediatric patients with severe TBI aged <16 years; (2) studies directly comparing ICP monitoring vs. no ICP monitoring; (3) studies reporting at least one predefined outcome: in-hospital mortality, complications, length of hospital stay, length of ICU stay, or mechanical ventilation duration; (4) RCT or cohort studies.

Exclusion criteria: (1) studies involving patients with coexisting neurological disorders (e.g., epilepsy, congenital brain malformations) alongside TBI that might confound outcomes; (2) studies involving pediatric patients with mild or moderate TBI as the primary population; (3) non-English studies.

### 2.3 Data extraction

Data extraction was performed independently by two reviewers, with any discrepancies resolved through consultation with a third reviewer. The extracted information included study characteristics, participant demographics, and outcomes. Study characteristics encompassed the first author, year of publication, study design, study location, and sample size. Participant demographics included age, gender distribution, and trauma severity assessed using the Injury Severity Score (ISS) or GCS score.

The primary outcomes of the meta-analysis were in-hospital mortality and complications. Complications were classified into six categories: infection-related complications (such as pneumonia, sepsis, and urinary tract infections), respiratory complications (such as acute respiratory distress syndrome and pulmonary insufficiency), renal dysfunction (such as acute kidney injury), cardiovascular events (such as cardiac arrest and hypotension), thromboembolic events (such as deep vein thrombosis and pulmonary embolism), and seizures. Secondary outcomes included the rate of craniotomy or craniectomy, length of hospital stay (in days), length of ICU stay (in days), duration of mechanical ventilation (in days), and medical costs (expressed in thousands of dollars).

### 2.4 Quality assessment

The quality of the included cohort studies was independently evaluated by two reviewers using the Methodological Index for Non-Randomized Studies (MINORS) (23). Discrepancies in judgments were resolved through discussion with a third investigator.

### 2.5 Statistical analysis

Meta-analyses were conducted using Review Manager (RevMan) version 5.4.1 (The Cochrane Collaboration, Oxford, UK). Continuous outcomes were reported as Weighted Mean Differences (WMD) with 95% Confidence Intervals (CIs), and dichotomous outcomes as pooled Odds Ratios (ORs) with 95% CIs. Heterogeneity was assessed using Cochrane's *Q* and the *I*^2^ statistic. A fixed-effects model was applied for *I*^2^ <50%, while a random-effects model was used for *I*^2^ ≥50%. Forest plots illustrated pooled effect sizes, and statistical significance was set at *p* < 0.05. Subgroup analyses compared studies using propensity score matching (PSM) with those that did not. Publication bias was not assessed due to the inclusion of fewer than 10 studies, following Cochrane Handbook guidelines.

## 3 Results

### 3.1 Study selection and characteristics

**Figure 1:**
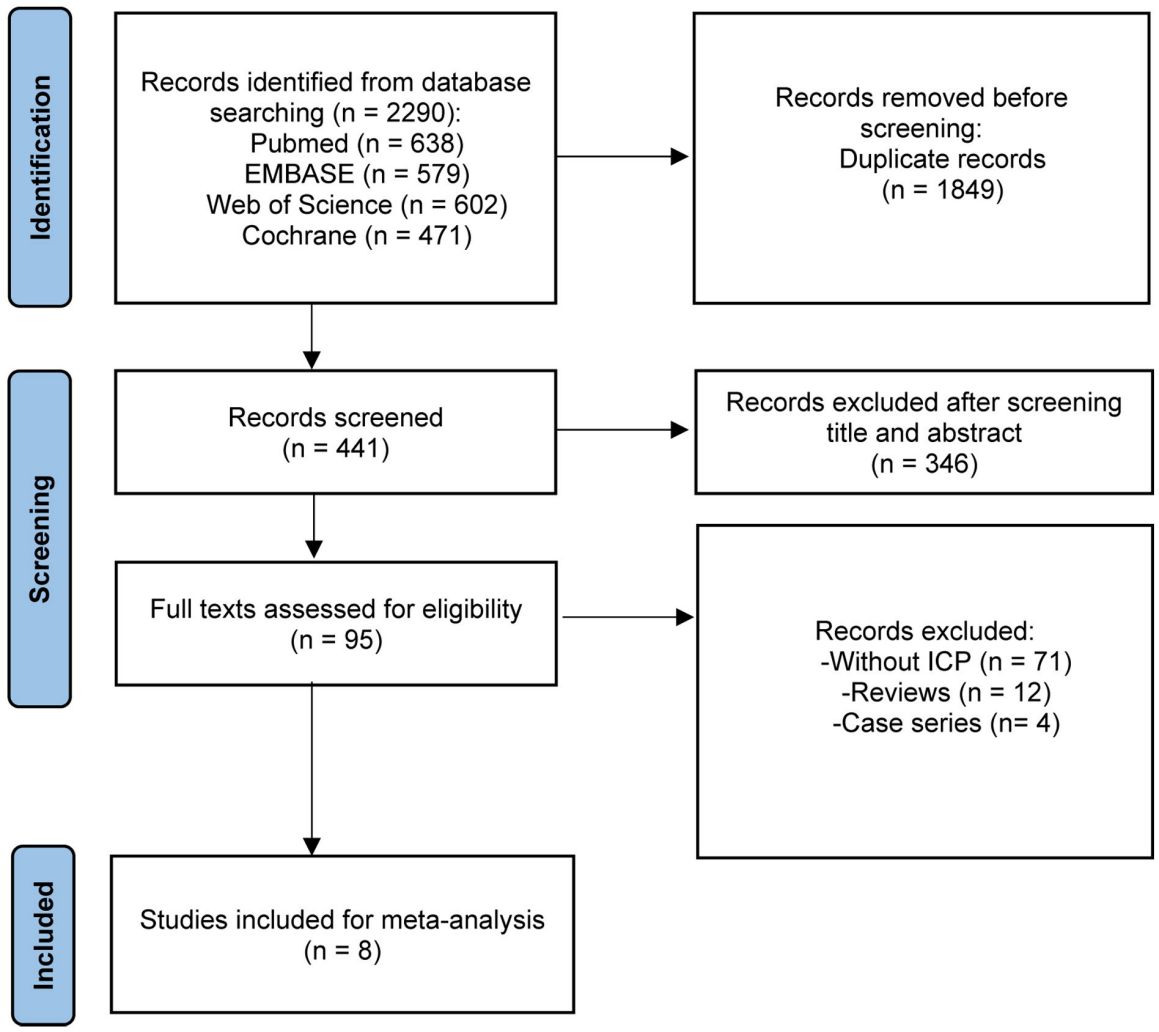
PRISMA flow chart of literature retrieval.

The initial search across PubMed, EMBASE, The Cochrane Library, and Web of Science identified 2,290 studies. After removing 1,849 duplicates, 441 publications were retained for title and abstract screening. Following this process, 346 studies were excluded, leaving 95 studies for full-text and reference review. Of these, 87 studies were further excluded, resulting in a final inclusion of 8 studies (Figure 1) (13–20). The characteristics of these studies are summarized in Table 1.

**Table 1:** Characteristics of the included studies.

**First author**	**Year**	**Study design**	**Country**	**Patients**, ***n***		**Age**, ***y*** **[Mean** ±**SD or Median (IQR)]**		**Sex (M/F)**		**ISS at admission[Mean** ±**SD or Median (IQR)]**		**GCS at admission [Mean** ±**SD or Median (IQR)]**	
				**ICP**+	**ICP–**	**ICP**+	**ICP–**	**ICP**+	**ICP–**	**ICP**+	**ICP–**	**ICP**+	**ICP–**
Salim	2008	Single center RCS	USA	32	97	7.0 ± 3.8	7.2 ± 4.2	23/9	70/27	25 ± 9^a^	18 ± 11^a^	4.7 ± 1.8	5.1 ± 2.0
Alkhoury	2014	Multicenter RCS	USA	283	2,817	8.8 ± 5.9	8.4 ± 6.0	166/117	1,762/1,055	31.5 ± 12.7^a^	27.6 ± 12.6^a^	3: 60.8%	3: 61.4%
												4: 7.8%	4: 5.0%
												5: 10.6%	5: 4.8%
												6: 8.1%	6: 9.1%
												7: 7.8%	7: 9.8%
												8: 4.9%	8: 9.9%
Alali	2015	Multicenter RCS	Canada	273	1,432	9 (11)	8 (11)	186/87	944/488	NR	NR	1 (2)	1 (1)
Arunkumar	2016	Single center PCS	India	30	20	7.4 ± 4.4	10.2 ± 4.9	18/12	13/7	NR	NR	4.4 ± 0.7	4.4 ± 1.3
Bennett	2017	Multicenter RCS	USA	1,002	2,082	7.5 (10.3)	6.8 (10)	642/360	1,314/768	27 ± 11^a^	20 ± 12^a^	3: 66.9%	3: 62.4%
												4: 5.0%	4: 3.6%
												5: 5.0%	5: 3.4%
												6: 10.4%	6: 11.4%
												7: 7.7%	7: 9.4%
												8: 5.0%	8: 9.8%
Banik	2019	Single center RCS	India	23	38	5 ± 2	5 ± 3	15/8	27/11	NR	NR	3-5: 34.8%	3-5: 26.3%
												6-8: 65.2%	6-8: 73.7%
Delaplain	2020	Multicenter RCS	USA	685	3,123	8 (10)	4 (11)	459/226	1,929/1,194	26 (13)^a^	16.0 (15)^a^	NR	NR
Shibahashi	2023	Multicenter RCS	Japan	210	840	11 (10)	11 (11)	146/64	603/237	12 (8)^b^	11 (7)^b^	JCS 100: 28.6%^c^	JCS 100: 27.9%^c^
												JCS 200: 36.2%^c^	JCS 200: 38.1%^c^
												JCS 300: 35.2%^c^	JCS 300: 34.0%^c^

RCS, retrospective cohort study; PCS, prospective cohort study; SD, standard deviation; IQR, interquartile range; M, male; F, female; ISS, Injury Severity Score; GCS, Glasgow Coma Scale; JCS, Japan Coma Scale; NR, not reported.

^a^There was statistically significant difference between the two groups. ^b^This study reported International Classification of Diseases 10th Revision–based injury severity score (ICISS). ^*C*^JCS 100–300 corresponds to a GCS score of 3–8, with higher scores indicating greater severity.

### 3.2 Risk of bias

The quality of the included studies was assessed using the MINORS criteria, as shown in Table 2. The main limitations were related to the retrospective design of most studies, including the lack of prospective data collection and sample size calculation. Overall, the quality of the included studies was considered moderate.

**Table 2:** Evaluation of quality of the included studies using the MINORS criteria.

**First author**	**Year**	**A clearly stated aim**	**Inclusion of consecutive patients**	**Prospective collection of data**	**End points appropriate to the aim of the study**	**Unbiased assessment of the study end point**	**Follow-up period appropriate to the aim of the study**	**Loss to follow-up <5%**	**Prospective calculation of the study size**.	**Total**
Salim	2008	2	2	0	2	1	2	2	0	11
Alkhoury	2014	2	2	0	2	1	2	2	0	11
Alali	2015	2	1	0	2	1	2	2	0	10
Arunkumar	2016	2	2	2	2	1	2	2	0	13
Bennett	2017	2	2	0	2	1	2	2	0	11
Banik	2019	2	1	0	2	1	2	2	0	10
Delaplain	2020	2	2	0	2	1	2	2	0	11
Shibahashi	2023	2	2	0	2	1	2	2	0	11

MINORS, Methodological Index for Non-Randomized Studies. The MINORS criteria are scored as follows: 0 points if not reported, 1 point if reported but inadequate, and 2 points if reported and adequate. The highest possible score is 16.

### 3.3 In-hospital mortality

**Figure 2:**
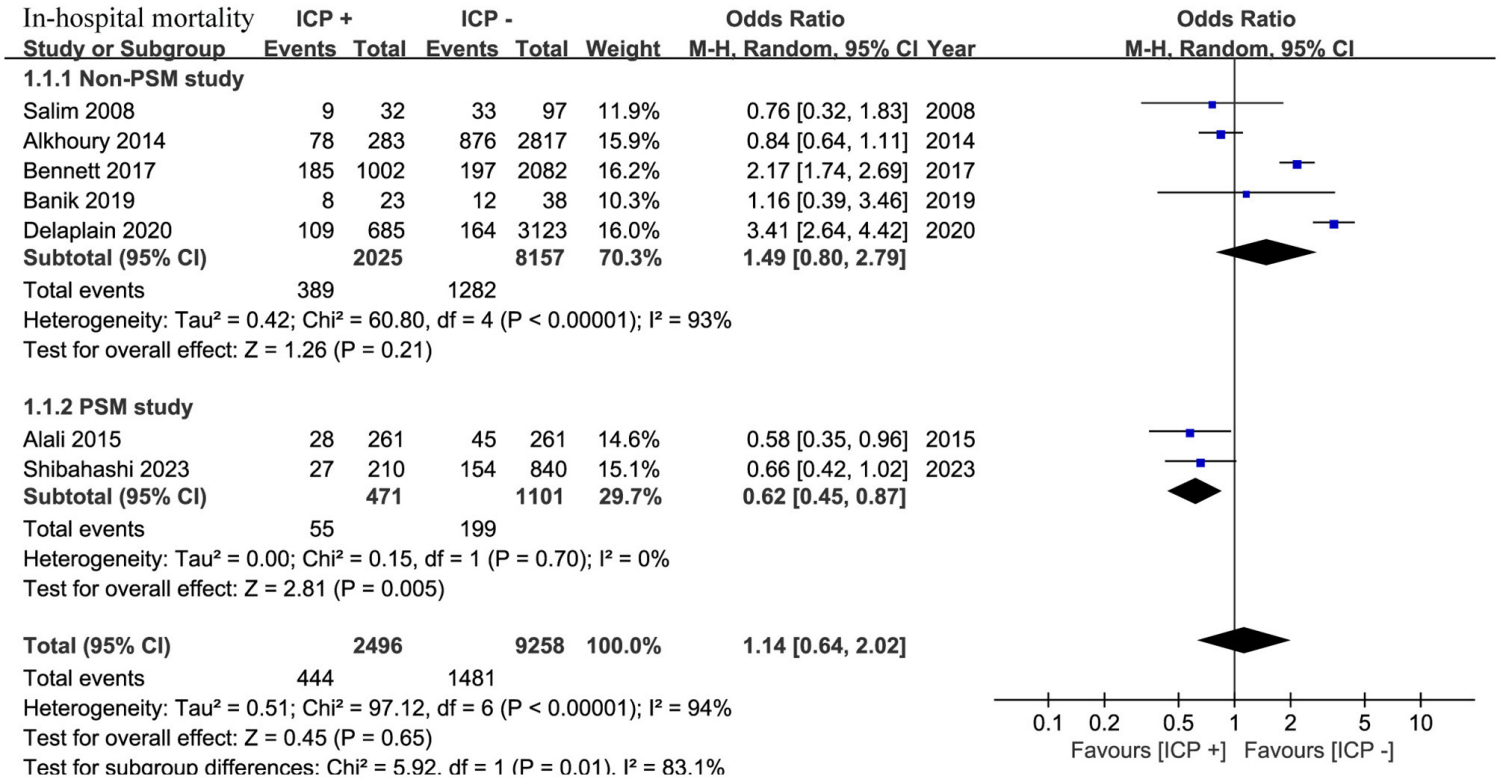
Meta-analysis of in-hospital mortality.

A total of seven studies (13, 15–20) involving 11,754 patients showed no significant difference in in-hospital mortality between the ICP monitoring and non-ICP monitoring groups (OR, 1.14; 95% CI, 0.64–2.02; *I*^2^ = 94%; *p* = 0.65) (Figure 2).

Subgroup analysis based on study design showed that in five non-PSM studies (16–20) with 10,182 patients, mortality was similar between the two groups (OR, 1.49; 95% CI, 0.80 to 2.79; *I*^2^ = 93%; *p* = 0.21). In two PSM studies (13, 15) with 1,572 patients, the ICP monitoring group had significantly lower in-hospital mortality compared to the non-ICP monitoring group (OR, 0.62; 95% CI, 0.45 to 0.87; *I*^2^ = 0%; *p* = 0.005) (Figure 2).

### 3.4 Complications

**Figure 3:**
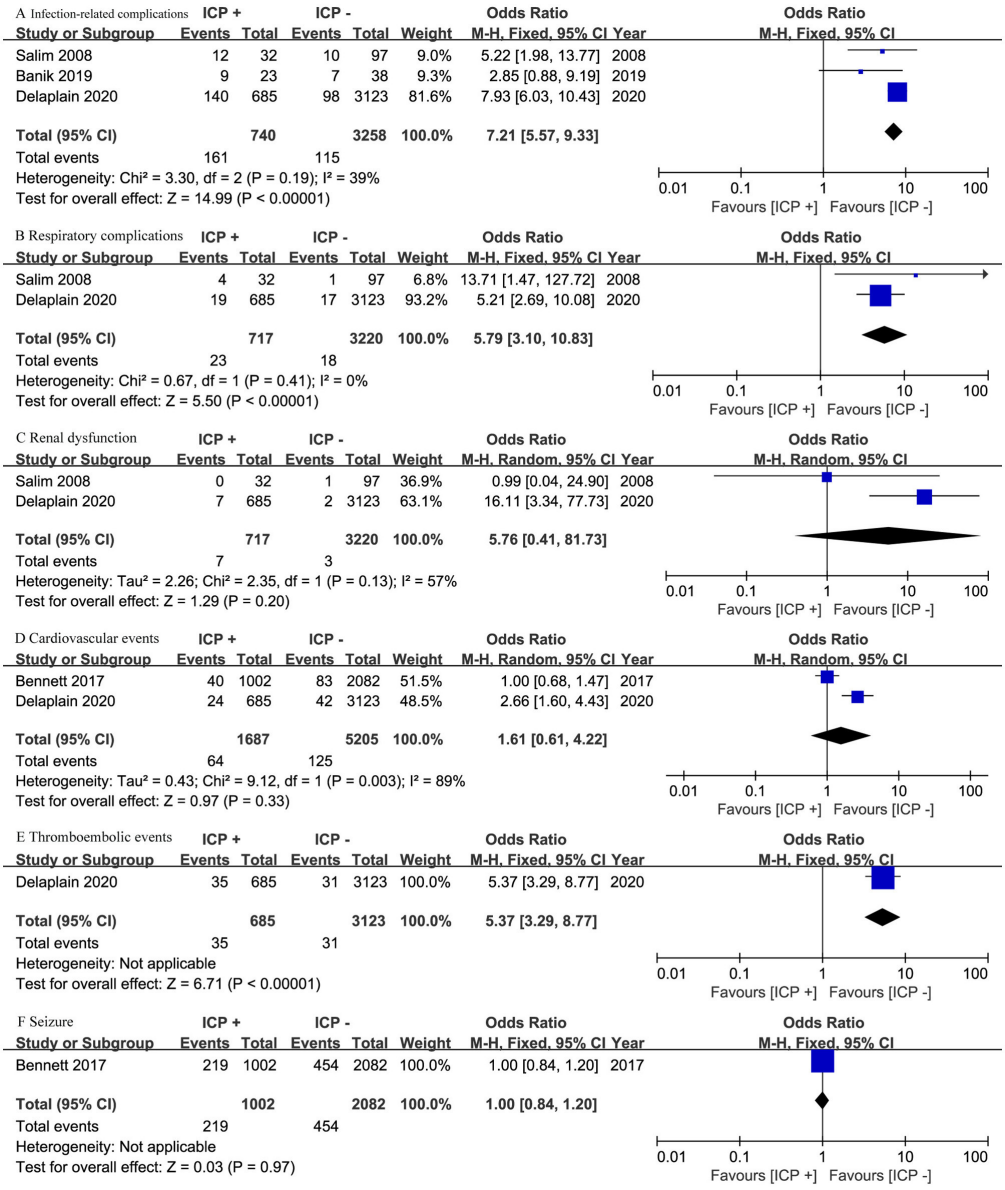
Meta-analysis of complications: **(A)** infection-related complications, **(B)** respiratory complications, **(C)** renal dysfunction, **(D)** cardiovascular events, **(E)** thromboembolic events, and **(F)** seizures.

Three studies (17, 19, 20) involving 3,998 patients reported infection-related complications, while two studies (19, 20) with 3,937 patients focused on respiratory complications and renal dysfunction. The ICP monitoring group had significantly higher risks of infection-related complications (OR, 7.21; 95% CI, 5.57–9.33; *I*^2^ = 39%; *p* < 0.001) and respiratory complications (OR, 5.79; 95% CI, 3.10 to 10.83; *I*^2^ = 0%; *p* < 0.001) (Figures 3A, B). However, there was no significant difference in renal dysfunction between the two groups (OR, 5.76; 95% CI, 0.41–81.73; *I*^2^ = 57%; *p* = 0.20) (Figure 3C).

Two studies (18, 19) involving 6,887 patients reported cardiovascular events, showing no significant difference between the groups (OR, 1.61; 95% CI, 0.61–4.22; *I*^2^ = 89%; *p* = 0.33) (Figure 3D). One study (19) with 3,808 patients reported thromboembolic events, indicating a higher risk in the ICP monitoring group (OR, 5.37; 95% CI, 3.29–8.77; *p* < 0.001) (Figure 3E). Another study (18) with 3,084 patients reported seizure events, showing no significant difference between the groups (OR, 1.00; 95% CI, 0.84–1.20; *p* = 0.97) (Figure 3F).

### 3.5 Rate of craniotomy or craniectomy

**Figure 4:**
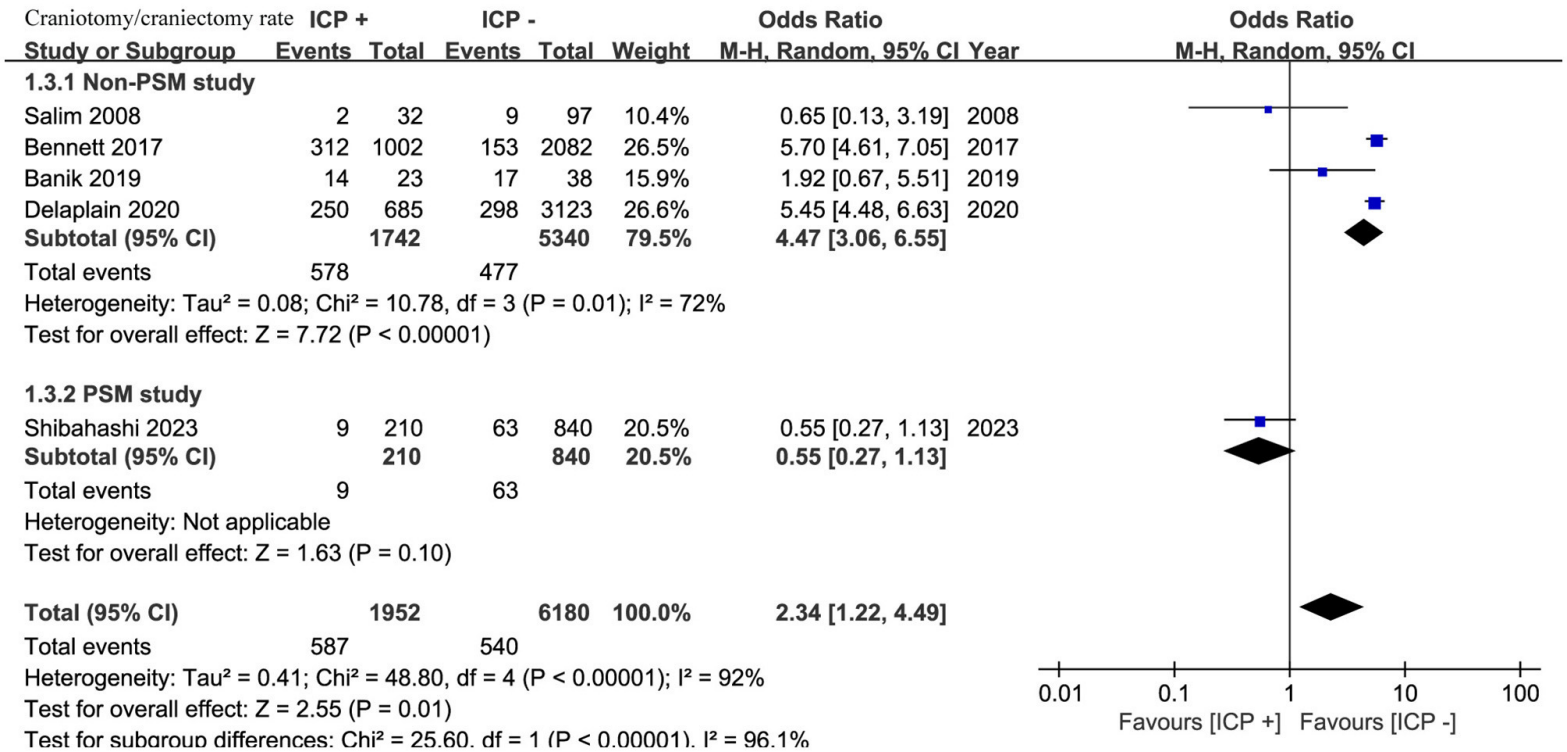
Meta-analysis of rate of craniotomy or craniectomy.

Five studies (15, 17–20) involving 8,132 patients showed a higher rate of craniotomy or craniectomy in the ICP monitoring group (OR, 2.34; 95% CI, 1.22–4.49; *I*^2^ = 92%; *p* = 0.01) (Figure 4).

Subgroup analysis by study design revealed that in four non-PSM studies (17–20) with 7,082 patients, the ICP monitoring group had a significantly higher rate of craniotomy or craniectomy (OR, 4.47; 95% CI, 3.06–6.55; *I*^2^ = 72%; *p* < 0.001). In one PSM study (15) with 1,050 patients, there was no significant difference between the groups (OR, 0.55; 95% CI, 0.27–1.13; *p* = 0.10) (Figure 4).

### 3.6 Length of stay

**Figure 5:**
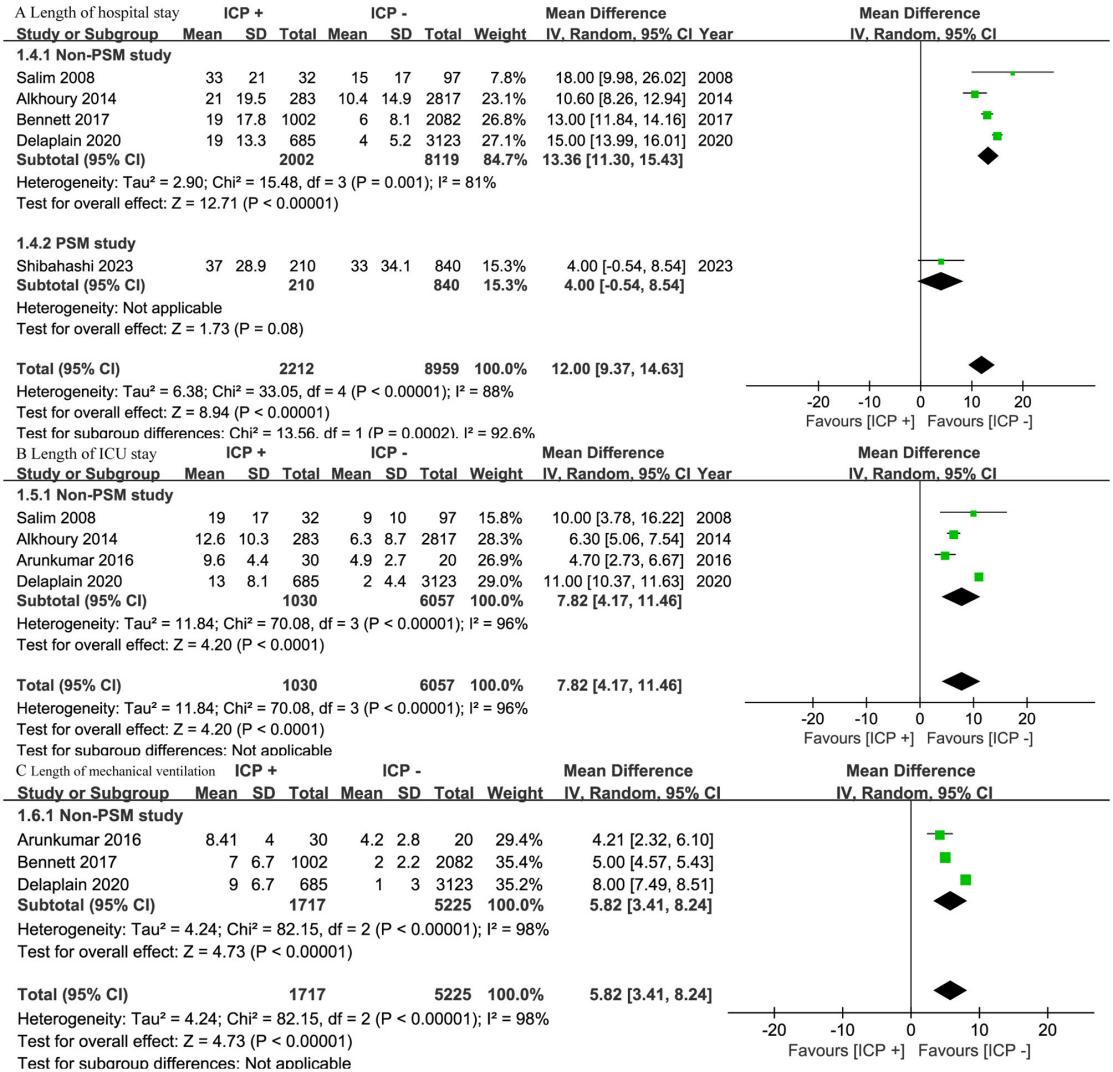
Meta-analysis of length of stays: **(A)** length of hospital stay, **(B)** length of ICU stay, and **(C)** length of mechanical ventilation.

Five studies (15, 16, 18–20) involving 11,171 patients found a longer hospital stay in the ICP monitoring group (OR, 12.00; 95% CI, 9.37–14.63; *I*^2^=88%; *p* < 0.001) (Figure 5). Subgroup analysis revealed that in four non-PSM studies (16, 18–20) with 10,121 patients, the ICP monitoring group had a significantly longer hospital stay (OR, 13.36; 95% CI, 11.30–15.43; *I*^2^ = 81%; *p* < 0.001). In one PSM study (15) with 1,050 patients, no significant difference was found (OR, 4.00; 95% CI, −0.54 to 8.54; *p* = 0.08) (Figure 5).

Four studies (14, 16, 19, 20) with 7,087 patients and three studies (14, 18, 19) with 6,942 patients showed that the ICP monitoring group had a longer ICU stay (OR, 7.82; 95% CI, 4.17 to 11.46; *I*^2^=96%; P < 0.001) and a longer duration of mechanical ventilation (OR, 5.82; 95% CI, 3.41–8.24; I^2^ = 98%; P < 0.001) (Figure 5). Subgroup analysis was not performed, as all studies were non-PSM.

### 3.7 Medical costs

**Figure 6:**
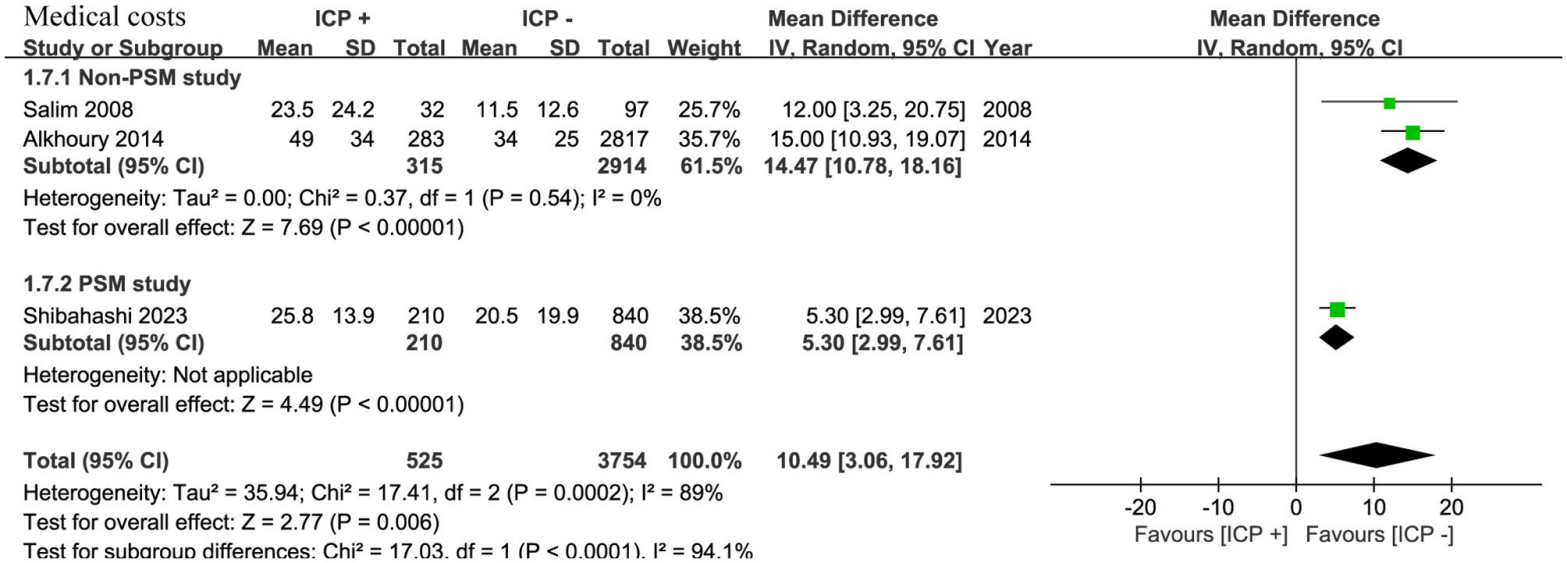
Meta-analysis of medical costs.

Three studies (15, 16, 20) involving 4,279 patients showed higher medical costs in the ICP monitoring group (WMD, 10.49; 95% CI, 3.06–17.92; *I*^2^ = 89%; *p* = 0.006) (Figure 6).

Subgroup analysis revealed that in two non-PSM studies (16, 20) with 2,917 patients, the ICP monitoring group had significantly higher medical costs (WMD, 14.47; 95% CI, 10.78–18.16; *I*^2^ = 0%; *p* < 0.001). In one PSM study (15) with 1,050 patients, the ICP monitoring group also had significantly higher costs (WMD, 5.30; 95% CI, 2.99–7.61; *p* < 0.001) (Figure 6).

## 4 Discussion

To evaluate the effects of ICP monitoring in pediatric severe TBI, this meta-analysis included eight studies with 12,987 patients. The main findings were that ICP monitoring did not significantly decrease overall in-hospital mortality. However, subgroup analysis revealed that, in PSM studies, ICP monitoring was associated with a lower in-hospital mortality rate. Additionally, ICP monitoring was linked to higher risks of infection-related and respiratory complications, thromboembolic events, a higher rate of craniotomy or craniectomy, longer hospital and ICU stays, longer durations of mechanical ventilation, and higher medical costs.

To our best knowledge, this is the first meta-analysis to evaluate the effects of ICP monitoring in children with severe TBI. Two prior meta-analyses (24, 25), focusing on adults with TBI, revealed consistent findings. The study published in 2014 (25), involving 11,038 patients, found no significant reduction in mortality with ICP monitoring but reported longer hospital and ICU stays. The 2016 study (24) involving 25,229 patients found no overall benefit of ICP monitoring. Similar to these findings, our study also showed no overall mortality reduction, with longer hospital and ICU stays in pediatric patients. However, different from them, our subgroup analysis in PSM studies indicated a survival benefit in children undergoing ICP monitoring. Notably, children and adults exhibit distinct characteristics in ICP management, including treatment thresholds of 20 mmHg for children and 22 mmHg for adults (7, 11). Studies further suggest that children have a lower tolerance for elevated ICP, with significantly shorter durations of safe exposure compared to adults (26), highlighting the necessity for pediatric-specific research.

While the overall meta-analysis showed no significant difference in in-hospital mortality, the subgroup analysis revealed a mortality reduction with ICP monitoring in PSM studies. The discrepancies between the overall meta-analysis and the subgroup analysis largely stem from differences in injury severity between the ICP-monitored and non-monitored groups in the included studies. In the unmatched studies, patients in the ICP-monitored group typically had more severe traumatic brain injuries, as evidenced by higher ISS and lower GCS scores. Similarly, in unmatched studies that often lack detailed treatment data, the elevated rates of craniotomy or craniectomy in the ICP-monitored group may reflect the greater injury severity rather than the impact of monitoring itself. In contrast, the sole PSM study (15) that adjusted for severity reported lower craniotomy or craniectomy rates in the ICP-monitored group, attributing this to ICP-guided management. These findings strongly suggest that the inconsistencies in outcomes are primarily a consequence of baseline differences in injury severity in the unmatched studies.

Extending the analysis to length of hospital stay and medical costs, a consistent trend was observed across studies. Although only one PSM study (15) reported these outcomes, its findings were consistent with those of unmatched studies, suggesting that ICP monitoring is associated with longer hospital stays and higher medical costs. Notably, the differences between groups in the PSM study were less pronounced compared to those in the unmatched studies. Similarly, unmatched studies consistently reported unfavorable outcomes associated with ICP monitoring, including higher rates of infection-related and respiratory complications, thromboembolic events, as well as prolonged ICU stays and ventilation durations. However, as previously discussed, it remains unclear whether these differences are directly attributable to ICP monitoring or are instead confounded by baseline imbalances, given that patients in the ICP-monitored group typically had more severe TBI.

The study by Shibahashi et al. (15) is particularly noteworthy as it stands out as one of the most methodologically rigorous investigations into ICP monitoring in children with severe TBI. This PSM analysis revealed that ICP monitoring significantly reduces in-hospital mortality, primarily through the implementation of aggressive and targeted ICP-guided treatments, which simultaneously reduced the rate of craniotomy or craniectomy in the ICP monitoring group. Notably, the study emphasizes that the effectiveness of ICP monitoring relies heavily on the interventions it enables, underscoring the importance of implementing ICP-guided treatment algorithms to achieve optimal outcomes (27–29). However, due to the inherent limitations of the study design, most of the original studies included in this analysis did not provide detailed information on whether ICP-guided treatments were consistently applied, which further impacts the reliability of the findings.

To address disparities in observational studies and clarify the true efficacy of ICP monitoring, we conducted a subgroup analysis distinguishing between PSM and non-PSM study outcomes. In this study, PSM studies demonstrated a significant link between ICP monitoring and reduced mortality, while non-PSM studies were confounded by unadjusted factors and fail to find significant differences between the two groups. Despite limited individual patient data precluding performing PSM on non-PSM studies, this analysis highlights PSM's vital role in refining outcome assessments. These findings resonate with the ongoing debate about the utility of ICP monitoring, which is further complicated by conflicting evidence from adult TBI studies. Notably, the SYNAPSE-ICU study (30), a large international prospective observational trial, provided critical insights into this controversy. Among 2,395 adult patients with acute brain injury, where 54% had TBI, ICP monitoring was associated with lower 6-month mortality in severe cases. Specifically, an adjusted hazard ratio of 0.35 was observed for patients with one or more unreactive pupils when aggressive ICP-guided therapies were implemented. This aligns with our PSM subgroup findings, indicating that ICP monitoring may benefit specific populations when confounding factors are minimized. The SYNAPSE-ICU study also highlights significant variability in monitoring practices across centers, with a median odds ratio of 4.50. This suggests that institutional protocols and therapeutic intensity, rather than monitoring alone, might drive differences in outcomes.

The BEST-TRIP trial (21), the only RCT comparing ICP-guided therapy with imaging or clinical examination in adult TBI, found no differences in mortality or functional outcomes. Critics note that its Latin American setting, where non-ICP management is routine, and its limited power may have influenced the results, yet it challenges the universal adoption of invasive monitoring. In pediatric populations, the absence of randomized trials and ethical concerns about withholding monitoring leave the field reliant on observational data. Both the SYNAPSE-ICU study and our analysis highlight that ICP monitoring's value lies in its integration into targeted treatment algorithms rather than mere data acquisition. For instance, SYNAPSE-ICU reported higher therapy intensity levels in monitored patients, correlating with improved survival, while our PSM subgroup showed lower craniotomy rates, suggesting optimized intervention timing. Despite these insights, unmatched observational studies risk confounding by indication, though propensity score-adjusted analyses and severity-based stratification suggest monitoring may reduce secondary injury in high-risk patients. However, risks such as longer ICU stays, infections, and costs require careful patient selection. Future pediatric studies should adopt standardized protocols to isolate monitoring's independent effects.

The studies included in our meta-analysis have several critical limitations. In addition to the previously discussed issues of inadequate baseline matching and insufficient reporting of ICP-guided interventions, other major flaws include the predominance of retrospective cohort studies, with only one small prospective cohort study (14). Consistent with previous literature (31–35), there were significant regional differences in ICP monitoring rates, with overall usage falling below the levels recommended by guidelines for the appropriate patient populations. Furthermore, treatment thresholds varied, with most studies lacking detailed reporting on thresholds. In addition, the application of brain tissue oxygen monitoring, which has been reported to improve pediatric outcomes when combined with ICP monitoring (36–38), was also not adequately addressed.

As a meta-analysis, our findings are limited by the lack of RCTs and high-quality prospective cohort studies, resulting in low-level evidence. Retrospective cohort studies dominate this analysis, introducing limitations due to inconsistent reporting of key admission severity indicators, which are vital for interpreting outcomes. Although available severity data are presented (Table 1), incomplete or missing metrics in some studies may confound our findings and weaken their validity. The retrospective design of the included studies, along with a focus on short-term outcomes and limited data on long-term recovery, further restricts the robustness and scope of our conclusions. These limitations underscore the need for prospective studies with standardized protocols and comprehensive outcome reporting to accurately assess the impact of ICP monitoring in pediatric severe TBI.

## 5 Conclusion

This meta-analysis found no overall reduction in in-hospital mortality with ICP monitoring in pediatric severe TBI, potentially due to baseline severity imbalances in retrospective studies. However, PSM studies suggest a mortality benefit, indicating that ICP monitoring may be effective when confounding is minimized. Increased complication risks, longer hospital/ICU stays, prolonged ventilation, and higher costs were associated with monitoring, though these may reflect injury severity rather than monitoring itself. Given the limitations of retrospective designs and variable severity reporting, these findings should be interpreted cautiously. High-quality prospective studies with standardized protocols are essential to confirm ICP monitoring's role in this population.

## Data Availability

The original contributions presented in the study are included in the article/supplementary material, further inquiries can be directed to the corresponding author.
